# Association of Adult Congenital Heart Disease With Pregnancy, Maternal, and Neonatal Outcomes

**DOI:** 10.1001/jamanetworkopen.2019.3667

**Published:** 2019-05-10

**Authors:** Kaylee Ramage, Kirsten Grabowska, Candice Silversides, Hude Quan, Amy Metcalfe

**Affiliations:** 1 Department of Community Health Sciences, University of Calgary, Calgary, Alberta, Canada; 2 Department of Obstetrics & Gynaecology, University of British Columbia, Vancouver, British Columbia, Canada; 3 Division of Cardiology, Department of Medicine, Toronto Congenital Cardiac Centre for Adults, University of Toronto, Toronto, Ontario, Canada; 4 Department of Medicine, University of Calgary, Calgary, Alberta, Canada; 5 Department of Obstetrics & Gynaecology, University of Calgary, Calgary, Alberta, Canada

## Abstract

**Importance:**

With the help of medical advances, more women with adult congenital heart disease (ACHD) are becoming pregnant. Adverse maternal, obstetric, and neonatal events occur more frequently in women with ACHD than in the general obstetric population. Adult congenital heart disease is heterogeneous, yet few studies have assessed whether maternal and neonatal outcomes differ across ACHD subtypes.

**Objective:**

To assess the association of ACHD and its subtypes with pregnancy, maternal, and neonatal outcomes.

**Design, Setting, and Participants:**

This cross-sectional study used data from the Discharge Abstract Database, which contains information on all hospitalizations in Canada (except Quebec) from fiscal years 2001-2002 through 2014-2015. Discharge Abstract Database information was linked with maternal and infant hospital records across Canada. All women who gave birth in hospitals during the study period were included in the study. Data were analyzed from December 18, 2017, to March 22, 2019.

**Exposures:**

Women with ACHD were identified using diagnostic and procedural codes. Subtypes of ACHD were classified using the Anatomic and Clinical Classification of Congenital Heart Defects scheme.

**Main Outcomes and Measures:**

Primary outcomes were defined a priori and included severe maternal morbidity (measured using the Maternal Morbidity Outcomes Indicator), neonatal morbidity and mortality (measured using the Neonatal Adverse Outcomes Indicator), ischemic placental disease, preterm birth, congenital anomalies, and small-for-gestational-age births. Absolute and relative rates of each outcome were calculated overall and by ACHD subtype. Logistic regression using generalized estimating equations assessed crude and adjusted odds ratios (aORs) for each outcome in women with ACHD compared with women without ACHD after adjustment for comorbidities, mode of delivery, and study year.

**Results:**

The 2114 women with ACHD included in the analysis (mean [SD] age, 29.4 [5.7] years) had significantly higher odds of maternal morbidity (aOR, 2.7; 95% CI, 2.2-3.4) and neonatal morbidity and mortality (aOR, 1.8; 95% CI, 1.6-2.1) compared with women without ACHD (n = 2 682 451). Substantial variation was observed between women with different subtypes of ACHD. For example, the aORs of preterm birth (<37 weeks) varied from 0.4 (95% CI, 0.4-0.5) for women with anomalies of atrioventricular junctions and valves to 4.7 (95% CI, 2.9-7.5) for women with complex anomalies of atrioventricular connections.

**Conclusions and Relevance:**

These results suggest that women with different subtypes of ACHD are not uniformly at risk for adverse maternal and neonatal outcomes. Although some women with ACHD can potentially expect healthy pregnancies, it appears that clinical care should be modified to address the heightened risks of certain ACHD subtypes.

## Key Points

**Question:**

Do pregnancy, maternal, and neonatal outcomes differ in women with different types of adult congenital heart disease?

**Findings:**

In this large cross-sectional study of 2114 women with adult congenital heart disease in Canada, maternal and neonatal outcomes varied by subtype of maternal heart condition. Adult congenital heart disease was associated with increased odds of adverse maternal, neonatal, and pregnancy outcomes during labor and delivery hospitalization.

**Meaning:**

These findings may help to highlight the importance of understanding and considering subtypes of adult congenital heart disease in clinical management of these high-risk pregnancies.

## Teaser

This cross-sectional study assesses the association of adult congenital heart disease and its subtypes among Canadian women with pregnancy and maternal and neonatal outcomes.

## Introduction

With the help of medical advances, more women with adult congenital heart disease (ACHD) are surviving to childbearing age. In the present era, adults represent two-thirds of the population with congenital heart disease in Canada^1^; ACHD affects 6.1 (95% CI, 5.7-6.6) per 1000 adults, almost 60% of whom are women.^2^ Consequently, more women with corrected, palliated, or uncorrected ACHD experience pregnancy.^3,4,5^ Pregnancy induces changes in cardiovascular hemodynamics that are generally well tolerated. However, the reversible but prolonged hemodynamic stress of pregnancy can have negative effects on the diseased heart; the hemodynamic and hormonal changes of pregnancy can increase the risk of arrhythmias, whereas the prothombotic state of pregnancy contributes to increased thromboembolic complications during pregnancy.^3,4^ Thus, in women with ACHD, these changes can worsen maternal cardiac function, increasing the risk of significant morbidity and, in rare cases, death of the mother or the developing fetus.^6^ Although pregnancy is often tolerated and well managed in women with ACHD,^7^ a systematic review involving 2491 pregnancies in women with ACHD^8^ found that adverse maternal, obstetric, and neonatal events occur at a higher rate than in the general obstetric population.^9,10^

Maternal ACHD is associated with a higher risk of pregnancy complications, including emergency cesarean delivery, postpartum hemorrhage, and cardiac complications. Furthermore, ACHD is associated with a higher risk of maternal morbidity and mortality^11^ and a higher risk of preterm birth, small-for-gestational-age (SGA) birth weight, and perinatal mortality.^12,13^ Several large cohort studies^11,14,15,16^ have examined risk factors for adverse outcomes in pregnancy for women with cardiac disease. For example, the Cardiac Disease in Pregnancy (CARPREG) Study (in Canada)^11,14^ found cardiac complications in as many as 16% of pregnancies and neonatal complications in 20% of pregnancies among women with heart disease. The multinational Registry of Pregnancy and Cardiac Disease (ROPAC) study^15^ found high rates of fetal and neonatal mortality in infants born to women with heart disease, as well as an increased rate of maternal mortality (1% compared with 0.007% in the general population). Finally, in 1302 complete pregnancies in women with ACHD, the Zwangerschap bij Aangeboren Hartafwijkingen I (ZAHARA I) study (in Belgium)^16^ found no association between maternal characteristics and adverse obstetric outcomes but found an association between ACHD and neonatal complications, including preterm birth, SGA birth weight, and neonatal mortality.

Although previous studies have demonstrated increased obstetric and neonatal risks for women with ACHD, most only included patients seen in tertiary care centers,^7,9,10,11,13,16,17^ combined women with structural and acquired heart disease,^11,13,14,15^ or lacked a control group.^7,9,10,11,14,16,17^ In Canada, only 23% of patients with ACHD are regularly followed up by physicians at a specialized ACHD clinic; therefore, previous studies may not be generalizable to current practice.^18^ In addition, previous studies have lacked the power to examine outcomes by ACHD subtype to determine whether all patients are uniformly at increased risk of adverse obstetric or neonatal outcomes.^10^ Thus, the current cross-sectional study used longitudinal administrative data from a nationwide sample to examine the association between subtypes of ACHD and maternal and neonatal outcomes.

## Methods

### Study Population

We conducted a cross-sectional study using population-level administrative data from the Discharge Abstract Database (DAD). The DAD is maintained by the Canadian Institute for Health Information and contains information on all hospitalizations in Canada (except Quebec) including those related to pregnancy, childbirth, and adverse medical events. Given that the DAD represents data from all hospitalizations in each province, there is no sampling of discharges. Data from the province of Quebec were unavailable because health care professionals in Quebec are not required to report data from acute care facilities to the Canadian Institute for Health Information. Ethics approval was obtained from the Conjoint Health Research Ethics Board of the University of Calgary, Calgary, Alberta, Canada, which granted a waiver of consent, given the deidentified nature of the administrative data used for the study. This study followed the Strengthening the Reporting of Observational Studies in Epidemiology (STROBE) reporting guideline for cross-sectional studies.

Maternal and infant records were linked by a unique identifier. An estimated 7.3% (95% CI, 7.2%-7.3%) of infant records could not be matched with their maternal identifier. These data were excluded from analysis. The DAD includes data on patient characteristics, admission and discharge dates, and as many as 25 diagnoses and 15 procedural codes associated with each hospitalization. Since fiscal year 2003-2004, diagnoses and procedures were coded using the *International Statistical Classification of Diseases and Related Health Problems, Tenth Revision, Canada* (*ICD-10-CA*), and the *Canadian Classification of Health Interventions*, respectively. Previously diagnoses were coded using the *International Classification of Diseases, Ninth Revision, Clinical Modification*, and the *Canadian Classification of Procedures*. Perinatal data within the DAD have been validated by Joseph and Fahey,^19^ whose results indicated high accuracy of the DAD and supported its use for perinatal surveillance and research when used with appropriate clinical inference.

The data included all women who delivered a live or stillborn infant in Canada (excluding Quebec) from April 1, 2001, to March 30, 2015. Women with ACHD were identified by searching the diagnostic coding positions for a code of interest (*ICD-10-CA* codes Q20-Q26). Information on the severity of ACHDs is not available in the DAD; thus, we classified ACHDs according to the Anatomic and Clinical Classification of Congenital Heart Defects scheme,^20^ which groups lesions into 10 categories (eTable 1 in the Supplement) mapped onto *International Statistical Classification of Diseases and Related Health Problems, Tenth Revision*, codes and was designed to permit common classification of congenital heart defects in epidemiological studies.^20^ Women without any form of ACHD were used as a control group in the analysis. Women with other forms of ACHD related to genetic conditions (ie, Marfan or Turner syndrome) were excluded from this analysis.

Birth events were identified using a validated diagnostic algorithm^19^ and classified as live or stillbirths. Although data on maternal and neonatal readmission are available in the DAD, our team only had access to readmission data for women with ACHD. Pregnancies ending before 20 weeks’ gestation were excluded. Mode of delivery was classified using a validated definition for cesarean and vaginal assisted deliveries, with birth events not listing a procedural code assumed to be delivered vaginally without assistance.^19^ Obstetric morbidity and mortality were identified using the Maternal Morbidity Outcome Indicator, which combines *ICD-10-CA*–based diagnostic and procedural codes related to the occurrence or treatment of severe events (eTable 2 in the Supplement). The validated Maternal Morbidity Outcome Indicator has a positive predictive value of 94.6% (95% CI, 72.3%-99.9%) to identify severe adverse obstetric outcomes during the delivery hospitalization.^21^ Ischemic placental disease was identified by the presence of placental abruption, preeclampsia, or delivery of an SGA infant.^22^ Neonatal morbidity was identified using the Neonatal Adverse Outcomes Indicator,^23^ which combines *ICD-10-CA*–based diagnostic and procedural codes associated with the occurrence or treatment of severe events (eTable 3 in the Supplement). Preterm births (classified as delivery at <37 weeks’ gestation [encompassing all preterm births] or <32 weeks’ gestation) were identified using information on gestational age at delivery. Canadian reference standards were used to define SGA based on the infant’s sex, gestational age at birth, and birth weight.^24^ Perinatal mortality was identified using codes for neonatal death, intrauterine fetal death, and stillbirth. Major visible congenital anomalies at birth were identified using a validated case definition (eTable 4 in the Supplement).^25^ Maternal and neonatal mortality were restricted to deaths occurring during the delivery and/or birth hospitalization.

To control for other variables contributing to antenatal maternal comorbidities, we used the Obstetric Comorbidity Index,^26,27^ which was specifically developed and validated to estimate adverse maternal and obstetric events when using administrative data and has been demonstrated to be more accurate for pregnancy-associated morbidity than other comorbidity indices.^28,29^ The predictive value of the Obstetric Comorbidity Index during the labor and delivery hospitalization has been shown to equal that during the entire pregnancy.^27^ We also independently assessed several of the comorbidities within the index to provide a profile of maternal comorbidity among women with ACHD. As an extension of the Obstetric Comorbidity Index, we also examined unplanned hysterectomy, gestational diabetes, and vaginal birth after cesarean delivery. Unplanned hysterectomy was identified as any hysterectomy occurring during the labor and delivery hospitalization that was not attributable to identified gynecological cancer.

### Statistical Analysis

Data were analyzed from December 18, 2017, to March 22, 2019. For each outcome, the absolute risk per 1000 pregnancies for women with and without specific subtypes of ACHD was calculated. We used χ^2^ tests to determine whether differences existed between women with and without ACHD for each outcome. Data on events involving fewer than 5 individuals were suppressed to prevent residual disclosure, as per the requirements of the Canadian Institute for Health Information and research ethics policy at the University of Calgary. Multivariate logistic regression was used to determine the odds of each outcome for women with different subtypes of ACHD. All multivariate models were adjusted for maternal comorbidities using the Obstetric Comorbidity Index, mode of delivery, and study year. The inclusion of these covariates as confounders was decided a priori based on previous literature, availability of data, and perceived clinical importance. Generalized estimating equations, a method of analysis used to account for correlated observations through the robust estimation of SEs, were used to account for lack of independence because women could contribute multiple pregnancies to the analyses. We selected an exchangeable correlation structure to account for our assumption that any 2 observations within a cluster (ie, a woman who experienced >1 pregnancy during the study period) was equally correlated with but unrelated to other clusters and that these correlations were not affected by time between pregnancies (as an autoregressive structure would assume) because the period between pregnancies was generally short (ie, 2-3 years). This correlation definition was especially appropriate because we analyzed longitudinal data using a cross-sectional study design.^30^ We also conducted a sensitivity analysis with an alternative correlation structure (independent correlation) to assess whether our results were robust to this choice (eTable 5 in the Supplement). Two-sided *P* < .05 was used to indicate statistical significance in all statistical tests and modeling. For demographic characteristics, we also calculated effect size using the Cohen φ^2^ statistic. Time trends were measured using nonparametric tests for trend for maternal morbidity, neonatal morbidity, SGA (<10th percentile) births, and preterm births (<37 weeks’ gestation) overall and for ACHD and non-ACHD groups. All analyses were conducted using Stata SE, version 14 (StataCorp).

## Results

The study population included women who delivered a live or stillborn infant in Canada (excluding Quebec) from the fiscal years 2001-2002 through 2014-2015 (n = 2 684 565) (Table 1). A total of 2114 births were to women with ACHD (mean [SD] age, 29.4 [5.7] years). Women with ACHD were younger than the general obstetric population (mean [SD] age, 29.8 [5.6] years; *P* < .001) and had higher rates of having at least 2 maternal morbidities (14.3% [95% CI, 12.9%-15.9%] vs 5.1% [5.0%-5.1%]) and of being primiparous (46.4% [95% CI, 44.3%-48.5%] vs 34.2% [34.2%-34.3%]) compared with women without ACHD (n = 2 682 451). Women with ACHD were more likely have a cesarean delivery (37.8% [95% CI, 35.8%-39.9%] vs 28.0% [95% CI, 27.9%-28.0%]). No women with ACHD underwent a vaginal birth after a prior cesarean delivery.

**Table 1.** Demographic Characteristics for Births to Women in Canada From Fiscal Years 2001-2002 to 2014-2015^a^

Characteristic	Total Births, No. (N = 2 684 565)	Births, No. (%) [95% CI, %]		*P* Value	Cohen φ^2^
		Women Without ACHD (n = 2 682 451)	Women With ACHD (n = 2114)		
Maternal age, y					
<20	99 364	99 274 (3.7) [3.7-3.7]	90 (4.3) [3.5-5.2]	.001	0.0029
20-24	387 873	387 514 (14.4) [14.4-14.5]	359 (17.0) [15.4-18.6]		
25-29	757 866	757 265 (28.2) [28.2-28.3]	601 (28.4) [26.5-30.4]		
30-34	881 826	881 154 (32.8) [32.8-32.9]	672 (31.8) [29.8-33.8]		
35-39	456 033	455 719 (17.0) [16.9-17.0]	314 (14.9) [13.4-16.4]		
40-44	96 131	96 062 (3.6) [3.6-3.6]	69 (3.3) [2.6-4.1]		
≥45	5472	5463 (0.2) [0.2-0.2]	9 (0.4) [0.2-0.8]		
Maternal comorbidities^b^					
Obstetric Comorbidity Index^c^					
0	2 167 592	2 166 095 (80.8) [80.7-80.8]	1497 (70.8) [68.8-72.7]	<.001	0.012
1	380 870	380 556 (14.2) [14.1-14.2]	250 (14.9) [13.4-16.4]		
≥2	136 103	135 800 (5.1) [5.0-5.1]	303 (14.3) [12.9-15.9]		
Severe preeclampsia or eclampsia	35 671	35 630 (1.3) [1.3-1.3]	41 (1.9) [1.4-2.6]	.01	<0.0001
Mild or unspecified preeclampsia	3410	3405 (0.1) [0.1-0.1]	5 (0.2) [0.1-0.6]	.16	<0.0001
Placenta previa	16 796	16 777 (0.6) [0.6-0.6]	19 (0.9) [0.6-1.4]	.11	<0.0001
Congestive heart failure	321	301 (0.01) [0.01-0.01]	20 (0.9) [0.6-1.5]	<.001	0.0006
Hypertension					
Pulmonary	192	165 (0.006) [0.005-0.007]	27 (1.3) [0.9-1.9]	<.001	0.0018
Preexisting	17 169	17 118 (0.6) [0.6-0.6]	51 (2.4) [1.8-3.2]	<.001	<0.0001
Gestational	116 213	116 102 (4.3) [4.3-4.4]	111 (5.3) [4.4-6.3]	.04	<0.0001
Cardiac valvular disease	2541	2434 (0.09) [0.09-0.09]	107 (5.1) [4.2-6.1]	<.001	0.0020
Drug abuse	13 474	13 460 (0.5) [0.5-0.5]	14 (0.7) [0.4-1.1]	.30	<0.0001
Asthma	11 902	11 865 (0.4) [0.4-0.5]	37 (1.8) [1.3-2.4]	<.001	<0.0001
Previous cesarean delivery	285 988	285 747 (10.7) [10.6-10.7]	241 (11.4) [10.1-12.8]	.27	<0.0001
Unplanned hysterectomy	3928	3921 (0.1) [0.1-0.2]	7 (0.3) [0.2-0.7]	.03	<0.0001
Gestational diabetes	26 998	26 969 (1.0) [1.0-1.0]	29 (1.4) [1.0-2.0]	.09	<0.0001
Parity					
Primiparous	918 851	917 870 (34.2) [34.2-34.3]	981 (46.4) [44.3-48.5]	<.001	<0.0001
Multiparous	1 765 714	1 764 581 (65.8) [65.7-65.8]	1133 (53.6) [51.5-55.7]		
Mode of delivery					
Cesarean	751 170	750 370 (28.0) [27.9-28.0]	800 (37.8) [35.8-39.9]	<.001	0.0110
Vaginal	1 933 395	1 932 081 (72.0) [72.0-72.1]	1314 (62.2) [60.1-64.2]		

Abbreviation: ACHD, adult congenital heart disease.

^a^ Excludes Quebec.

^b^ Maternal comorbidities from the Obstetric Comorbidity Index with outcomes in fewer than 5 patients in either group are not included; however, these were considered in deriving the overall Obstetric Comorbidity Index.

^c^ Calculated as number of comorbidities.

A small proportion of women without ACHD had severe maternal morbidity (1.1%; 95% CI, 1.1%-1.1%) during their labor and delivery hospitalization (Table 2). The odds of severe maternal morbidity were nearly tripled for women with any type of ACHD, occurring in 4.0% (95% CI, 3.3%-4.9%; adjusted odds ratio [aOR], 2.7 [95% CI, 2.2-3.4]). Substantial variation in outcomes between women with different ACHD subtypes was observed. Women with anomalies of atrioventricular junctions and valves, functionally univentricular hearts, or anomalies of the extrapericardial arterial trunks did not have increased odds of severe maternal morbidity compared with the general obstetric population, whereas women with ventricular septal defects had the highest odds of experiencing severe maternal morbidity (aOR, 3.3; 95% CI, 2.0-5.5), which occurred in 4.7% (95% CI, 2.9%-7.5%) of women with this subtype of ACHD. Maternal morbidity did not vary by delivery type, with an aOR of 3.0 (95% CI, 2.1-4.4) in women with ACHD having a vaginal delivery and 2.7 (95% CI, 2.1-3.6) having a cesarean delivery. Maternal readmission within 90 days of delivery for women with ACHD was uncommon, including 3.3% (95% CI, 2.6%-4.2%) of all pregnancies. Severe maternal morbidity was more common in the initial hospitalization for this group of women (11.4%; 95% CI, 5.7%-21.4%) than in the general population of women with ACHD (4.0%; 95% CI, 3.3%-4.9%). When diagnoses and procedures occurring during readmissions were accounted for, the incidence of severe maternal morbidity increased to 30.0% (95% CI, 20.3%-41.9%). For women with ACHD, no time trend was observed for the incidence of severe maternal morbidity; however, for women without ACHD, severe maternal morbidity decreased during the study period from 1.3% (95% CI, 1.2%-1.4%) in fiscal year 2001-2002 to 1.0% (95% CI, 1.0%-1.1%) in fiscal year 2014-2015 (*P* = .04).

**Table 2.** Adverse Maternal Outcomes for Women With Different Forms of ACHD in Canada From Fiscal Years 2001-2002 to 2014-2015^a^

Participant Group	Total Births, No.	Outcome					
		Severe Maternal Morbidity			Ischemic Placental Disease		
		No. (%) [95% CI, %]	OR (95% CI)		No. (%) [95% CI, %]	OR (95% CI)	
			Crude	Adjusted^b^		Crude	Adjusted^b^
Women without ACHD	2 682 451	29 785 (1.1) [1.1-1.1]	1 [Reference]	1 [Reference]	384 811 (14.3) [14.3-14.4]	1 [Reference]	1 [Reference]
Women with any type of ACHD	2114	85 (4.0) [3.3-4.9]^c^	3.7 (3.0-4.6)^c^	2.7 (2.2-3.4)^c^	408 (19.3) [17.7-21.0]^c^	1.4 (1.3-1.6)^c^	1.0 (0.8-1.1)
Women with a subtype of ACHD							
Heterotaxy	42	NA^d^	NA^d^	NA^d^	10 (23.8) [13.2-39.1]	1.8 (0.9-3.8)	1.8 (0.8-4.1)
Anomalies of venous return	410	19 (4.6) [3.0-7.2)^c^	4.3 (2.7-6.8)^c^	3.1 (1.9-5.0)^c^	74 (18.0) [14.6-22.1]^c^	1.3 (1.0-1.7)^c^	0.6 (0.4-1.0)^c^
Anomalies of the atria and interatrial communications	404	17 (4.2) [2.6-6.7]^c^	3.9 (2.4-6.3)^c^	2.8 (1.7-4.6)^c^	73 (18.1) [14.6-22.1]^c^	1.3 (1.0-1.7)^c^	0.6 (0.4-1.0)^c^
Anomalies of atrioventricular junctions and valves	1440	33 (2.3) [1.6-3.2)^c^	2.1 (1.5-2.9)^c^	1.2 (0.8-1.6)	196 (13.6) [11.9-15.5]	1.0 (0.8-1.1)	0.1 (0.1-0.1)^c^
Complex anomalies of atrioventricular connections	98	NA^d^	NA^d^	NA^d^	24 (24.5) [17.0-34.0]	2.0 (1.2-3.1)^c^	2.0 (1.2-3.4)^c^
Functionally univentricular hearts	151	5 (3.3) [1.4-7.7]^c^	3.0 (1.2-7.4)^c^	2.2 (1.0-5.3)	37 (24.5) [18.3-32.0]	1.9 (1.3-2.8)^c^	1.9 (1.2-3.0)^c^
Ventricular septal defects	341	16 (4.7) [2.9-7.5]^c^	4.3 (2.6-7.2)^c^	3.3 (2.0-5.5)^c^	51 (15.0) [11.5-19.2]	1.0 (0.8-1.4)	0.8 (0.4-1.2)
Anomalies of the ventricular outflow tracts	949	32 (3.4) [2.4-4.7]^c^	3.1 (2.2-4.4)^c^	2.3 (1.6-3.2)^c^	190 (20.0) [17.6-22.7]	1.5 (1.3-1.8)^c^	1.1 (0.9-1.3)
Anomalies of the extrapericardial arterial trunks	413	15 (3.6) [2.2-5.9]	3.4 (2.0-5.6)^c^	2.4 (1.4-4.2)	84 (20.3) [16.7-24.5]	1.5 (1.2-1.9)^c^	1.1 (0.8-1.5)
Congenital anomalies of the coronary arteries	108	NA^d^	NA^d^	NA^d^	25 (23.1) [16.1-32.1]	1.8 (1.2-2.8)^c^	2.0 (1.2-3.3)^c^

Abbreviations: ACHD, adult congenital heart disease; NA, not applicable; OR, odds ratio.

^a^ Excludes Quebec.

^b^ Adjusted for Obstetric Comorbidity Index, mode of delivery, and year of birth.

^c^ *P* < .05 for comparison of women with and without ACHD and women with specific subtypes of ACHD and women without ACHD.

^d^ Sample size less than 5.

Overall, the odds of ischemic placental disease were not significantly different between women with and without ACHD. For some subtypes of ACHD, women with ACHD had significantly lower odds of ischemic placental disease compared with the general obstetric population (ie, anomalies of venous return [aOR, 0.6; 95% CI, 0.4-1.0], anomalies of the atria and interatrial communications [aOR, 0.6; 95% CI, 0.4-1.0], and anomalies of the atrioventricular junctions and valves [aOR, 0.1; 95% CI, 0.1-0.1]). Some subtypes were associated with significantly higher odds of ischemic placental disease compared with the general obstetric population, including congenital anomalies of the coronary arteries (aOR, 2.0; 95% CI, 1.2-3.3), functionally univentricular hearts (aOR, 1.9; 95% CI, 1.2-3.0), and complex anomalies of the atrioventricular connections (aOR, 2.0; 95% CI, 1.2-3.4).

Neonatal outcomes are summarized in Table 3. Overall, infants born to women with all subtypes of ACHD had significantly higher odds of severe neonatal morbidity (aOR, 1.8; 95% CI, 1.6-2.1) compared with infants born to women in the general population, occurring in 11.4% (95% CI, 10.1%-12.8%) of births. However, variation by subtype occurred. Infants born to mothers who had heterotaxy (aOR, 4.3; 95% CI, 1.9-9.5) or functionally univentricular hearts (aOR, 4.4; 95% CI, 2.9-6.6) had higher odds of severe neonatal morbidity, which occurred in more than 20% of births for each of these subtypes. Conversely, some subtypes of ACHD were not associated with substantially different results for neonatal morbidity, including anomalies of venous return, anomalies of the atria and interatrial communications, anomalies of the atrioventricular junctions and valves, and anomalies of the extrapericardial arterial trunks. Neonatal morbidity did not vary by delivery type for women with ACHD, with an aOR of 2.1 (95% CI, 1.7-2.6) for women with cesarean deliveries and 1.7 (95% CI, 1.4-2.1) for women with vaginal deliveries.

**Table 3.** Adverse Neonatal Outcomes for Infants Born to Women With Different Subtypes of ACHD in Canada From Fiscal Years 2001-2002 to 2014-2015^a^

Participant Group	Outcome								
	Severe Neonatal Morbidity and Mortality			Perinatal Mortality			Major Visible Congenital Anomalies		
	No. (%) [95% CI, %]	OR (95%CI)		No. (%) [95% CI, %]	OR (95%CI)		No. (%) [95% CI, %]	OR (95%CI)	
		Crude	Adjusted^b^		Crude	Adjusted^b^		Crude	Adjusted^b^
All infants of women without ACHD	140 307 (5.2) [5.2-5.3]	1 [Reference]	1 [Reference]	6593 (0.2) [0.2-0.3]	1 [Reference]	1 [Reference]	17 197 (0.6) [0.6-0.7]	1 [Reference]	1 [Reference]
All infants of women with any type of ACHD	241 (11.4) [10.1-12.8]^c^	2.3 (2.0-2.6)^c^	1.8 (1.6-2.1)^c^	17 (0.8) [0.5-1.3]^c^	3.2 (2.0-5.2)^c^	2.6 (1.6-4.3)^c^	27 (1.3) [0.9-1.9]^c^	2.0 (1.3-3.0)^c^	1.9 (1.3-2.8)^c^
Infants of women with a subtype of ACHD									
Heterotaxy	9 (21.4) [11.4-36.5]^c^	4.8 (2.3-10.1)^c^	4.3 (1.9-9.5)^c^	NA^d^	NA^d^	NA^d^	NA^d^	NA^d^	NA^d^
Anomalies of venous return	35 (8.5) [6.2-11.7]^c^	1.7 (1.2-2.4)^c^	1.2 (0.9-1.8)	NA^d^	NA^d^	NA^d^	7 (1.7) [0.8-3.5]^c^	2.7 (1.3-5.7)^c^	2.5 (1.2-5.2)^c^
Anomalies of the atria and interatrial communications	35 (8.7) [6.3-11.8]^c^	1.7 (1.2-2.4)^c^	1.3 (0.9-1.8)	NA^d^	NA^d^	NA^d^	7 (1.7) [8.3-3.6]^c^	2.7 (1.3-5.7)^c^	2.5 (1.2-5.3)^c^
Anomalies of atrioventricular junctions and valves	110 (7.6) [6.4-9.1]^c^	1.5 (1.2-1.8)^c^	0.9 (0.7-1.1)	7 (0.5) [0.2-1.0]	1.9 (0.9-4.1)	0.9 (0.4-2.0)	12 (0.8) [0.5-1.5]	1.3 (0.7-2.3)	1.1 (0.6-2.0)
Complex anomalies of atrioventricular connections	19 (19.4) [12.7-28.5]^c^	4.3 (2.6-7.2)^c^	4.1 (2.4-6.9)^c^	NA^d^	NA^d^	NA^d^	NA^d^	NA^d^	NA^d^
Functionally univentricular hearts	32 (21.2) [15.4-28.5]^c^	4.8 (3.2-7.1)^c^	4.4 (2.9-6.6)^c^	NA^d^	NA^d^	NA^d^	NA^d^	NA^d^	NA^d^
Ventricular septal defects	34 (10.0) [7.2-13.6]^c^	2.0 (1.4-2.9)^c^	1.7 (1.2-2.4)^c^	NA^d^	NA^d^	NA^d^	6 (1.8) [0.8-3.9]^c^	2.8 (1.2-6.2)^c^	2.6 (1.2-5.8)
Anomalies of the ventricular outflow tracts	113 (11.9) [10.0-14.1]^c^	2.4 (2.0-3.0)^c^	2.0 (1.6-2.4)^c^	5 (0.4) [0.2-1.3]	2.1 (0.9-5.2)	1.8 (0.7-4.4)	9 (0.9) [0.5-1.8]	1.5 (0.8-2.9)	1.4 (0.7-2.7)
Anomalies of the extrapericardial arterial trunks	37 (9,0) [6.6-12.1]^c^	1.8 (1.3-2.5)^c^	1.4 (1.0-2.0)	NA^d^	NA^d^	NA^d^	5 (1.2) [0.5-2.9]	1.9 (0.8-4.6)	1.8 (0.7-4.3)
Congenital anomalies of the coronary arteries	19 (17.6) [11.5-26.0]^c^	3.8 (2.3-6.2)^c^	3.8 (2.3-6.2)^c^	NA^d^	NA^d^	NA^d^	NA^d^	NA^d^	NA^d^

Abbreviations: ACHD, adult congenital heart disease; NA, not applicable; OR, odds ratio.

^a^ Excludes Quebec.

^b^ Adjusted for Obstetric Comorbidity Index, mode of delivery, and year of birth.

^c^ *P* < .05 for comparison of infants born to women with and without ACHD and infants born to women with specific subtypes of ACHD and women without ACHD.

^d^ Sample size less than 5.

This pattern was also noted when evaluating incidence of perinatal mortality. Although a small number of events precluded reporting on perinatal mortality for many ACHD subtypes, ACHD was associated with increased odds of perinatal mortality among infants born to women with any type of ACHD (aOR, 2.6; 95% CI, 1.6-4.3), occurring in 0.8% (95% CI, 0.5%-1.3%) of births compared with 0.2% (95% CI, 0.2%-0.3%) in the general population. As noted for neonatal morbidity, this risk was not uniform, and the odds of perinatal mortality among infants born to women with some subtypes of ACHD (ie, anomalies of atrioventricular junctions and valves and anomalies of atrioventricular flow tracts) did not substantially differ from those of the general population.

Overall, the odds of major visible congenital anomalies in infants born to mothers with ACHD compared with those born to women in the general population were significantly higher (aOR, 1.9; 95% CI, 1.3-2.8), occurring in 1.3% (95% CI, 0.9%-1.9%) of births. Results for several subtypes of ACHD did not significantly differ from those of the general population; however, infants born to women with anomalies of venous return (aOR, 2.5; 95% CI, 1.2-5.1) and anomalies of the atria and interatrial communications (aOR, 2.5; 95% CI, 1.2-5.3) had significantly higher odds of major visible congenital anomalies at birth.

The rates of preterm birth are summarized in Table 4. Preterm birth was more common among infants born to women with ACHD and ranged from 9.7% (95% CI, 7.0%-13.3%) in women with ventricular septal defects to 27.2% (95% CI, 20.6%-34.8%) in women with functionally univentricular hearts. Overall, preterm births (<37 weeks’ gestation) were associated with 1.4 (95% CI, 1.3-1.8) times higher odds among women with ACHD compared with those without ACHD (aOR, 1.5; 95% CI, 1.3-1.8), occurring in 13.9% of births (95% CI, 12.5%-15.4%) in women with ACHD. Women with ACHD also had higher odds of having a preterm birth at less than 32 weeks’ gestation (aOR, 1.7; 95% CI, 1.3-2.3), occurring in 3.1% of births (95% CI, 2.5%-4.0%). However, variation occurred between women with different subtypes of ACHD. Women with anomalies of the atria and interatrial communications, anomalies of venous return, or ventricular septal defects did not show a significant association with preterm birth before 37 or 32 weeks’ gestation. At gestation of less than 37 and less than 32 weeks, women with complex anomalies of atrioventricular connections (aORs, 4.7 [95% CI, 2.9-7.5] and 5.0 [95% CI, 2.0-12.2], respectively), functionally univentricular hearts (aORs, 4.6 [95% CI, 3.1-6.8] and 5.3 [95% CI, 2.4-11.4], respectively), and congenital anomalies of the coronary arteries (aORs, 4.2 [95% CI, 2.7-6.6] and 4.9 [95% CI, 2.1-11.3], respectively) had the highest odds of preterm birth. Women with anomalies of atrioventricular junctions and valves had reduced odds of preterm birth at less than 37 weeks (aOR, 0.4 [95% CI, 0.4-0.5]) and less than 32 weeks (aOR, 0.4 [95% CI, 0.2-0.6]) compared with women without ACHD.

**Table 4.** Preterm Birth and SGA Outcomes for Infants Born to Women in Canada From Fiscal Years 2001-2002 to 2014-2015 by ACHD Subtype^a^

Infant Group	Preterm Birth <37 wk			Preterm Birth <32 wk			SGA Births^b^		
	No. (%) [95% CI, %]	OR (95% CI)		No. (%) [95% CI, %]	OR (95% CI)		No. (%) [95% CI, %]	OR (95% CI)	
		Crude	Adjusted^c^		Crude	Adjusted^c^		Crude	Adjusted^c^
Infants of women without ACHD	199 447 (7.4) [7.4-7.5]	1 [Reference]	1 [Reference]	32 667 (1.2) [1.2-1.2]	1 [Reference]	1 [Reference]	230 895 (8.7) [8.7-8.8]	1 [Reference]	1 [Reference]
Infants of women with any type of ACHD	293 (13.9) [12.5-15.4]	2.0 (1.7-2.2)^d^	1.5 (1.3-1.8)^d^	66 (3.1) [2.5-4.0]^d^	2.6 (2.0-3.3)^d^	1.7 (1.3-2.3)^d^	269 (12.8) [11.5-14.3]^d^	1.5 (1.3-1.7)^d^	1.4 (1.2-1.6)^d^
Infants of women with a subtype of ACHD									
Heterotaxy	8 (19.0) [9.7-33.9]^d^	2.8 (1.3-6.2)^d^	2.7 (1.1-6.3)^d^	NA^e^	NA^e^	NA^e^	9 (22.0) [11.7-37.3]	2.9 (1.4-6.2)^d^	2.9 (1.3-6.1)^d^
Anomalies of venous return	51 (12.4) [9.6-16.0]^d^	1.7 (1.3-2.3)^d^	1.1 (0.8-1.6)	12 (2.9) [1.7-5.1]	2.4 (1.3-4.3)^d^	1.4 (0.8-2.6)	47 (11.5) [8.8-15.0]	1.3 (0.99-1.8)	1.2 (0.9-1.6)
Anomalies of the atria and interatrial communications	46 (11.4) [8.6-14.9]^d^	1.5 (1.1-2.1)^d^	1.0 (0.7-1.4)	12 (3.0) [1.7-5.2]^d^	2.4 (1.3-4.3)^d^	1.4 (0.8-2.6)	47 (11.7) [8.9-15.2]^d^	1.4 (1.0-1.9)^d^	1.2 (0.9-1.7)
Anomalies of atrioventricular junctions and valves	142 (9.9) [8.4-11.5]^d^	1.3 (1.1-1.6)^d^	0.4 (0.4-0.5)^d^	20 (1.4) [0.9-2.1]	1.1 (0.7-1.8)	0.4 (0.2-0.6)^d^	112 (7.9) [6.6-9.4]	0.9 (0.8-1.1)	0.7 (0.5-0.8)^d^
Complex anomalies of atrioventricular connections	26 (26.5) [18.7-36.2]^d^	4.4 (2.8-6.9)^d^	4.7 (2.9-7.5)^d^	6 (6.1) [2.8-13.0]^d^	5.3 (2.3-11.9)^d^	5.0 (2.0-12.2)^d^	15 (15.3) [9.4-23.9]^d^	1.9 (1.1-3.3)	1.9 (1.1-3.2)^d^
Functionally univentricular hearts	41 (27.2) [20.6-34.8]^d^	4.4 (3.1-6.4)^d^	4.6 (3.1-6.8)^d^	11 (7.3) [4.1-12.7]^d^	6.1 (3.3-11.4)^d^	5.3 (2.4-11.4)^d^	25 (16.6) [11.4-23.4]	2.1 (1.4-3.2)^d^	2.0 (1.3-3.1)^d^
Ventricular septal defects	33 (9.7) [7.0-13.3]	1.3 (0.9-1.9)	1.0 (0.7-1.5)	10 (2.9) [1.6-5.4]	2.4 (1.2-4.7)^d^	1.6 (0.7-3.4)	35 (10.3) [7.5-14.0]	1.2 (0.8-1.7)	1.1 (0.8-1.6)
Anomalies of the ventricular outflow tracts	140 (14.8) [12.6-17.2]^d^	2.1 (1.8-2.5)^d^	1.8 (1.4-2.1)^d^	29 (3.1) [2.1-4.4]^d^	2.5 (1.8-3.7)^d^	1.9 (1.3-2.8)^d^	112 (11.9) [10.0-14.1]	1.4 (1.2-1.7)^d^	1.3 (1.1-1.6)^d^
Anomalies of the extrapericardial arterial trunks	63 (15.3) [12.1-19.1]^d^	2.2 (1.7-2.9)^d^	1.8 (1.3-2.5)^d^	10 (2.4) [1.3-4.4]^d^	2.0 (1.0-3.7)^d^	1.4 (0.7-2.7)	57 (13.9) [10.9-17.6]	1.7 (1.2-2.2)^d^	1.5 (1.1-2.1)^d^
Congenital anomalies of the coronary arteries	26 (24.1) [16.9-33.1]^d^	3.8 (2.5-6.0)^d^	4.2 (2.7-6.6)^d^	6 (5.6) [2.4-11.9]	4.8 (2.1-10.8)^d^	4.9 (2.1-11.3)^d^	16 (14.8) [9.3-22.9]^d^	1.8 (1.1-3.1)^d^	1.8 (1.1-3.1)^d^

Abbreviations: ACHD, adult congenital heart disease; NA, not applicable; OR, odds ratio; SGA, small for gestational age.

^a^ Excludes Quebec.

^b^ Indicates less than tenth percentile.

^c^ Adjusted for Obstetric Comorbidity Index, mode of delivery, and year of birth.

^d^ *P* < .05 for comparison of infants born to women with and without ACHD and infants born to women with specific subtypes of ACHD and women without ACHD.

^e^ Sample size less than 5.

Overall, 12.8% (95% CI, 11.5%-14.3%) of women with ACHD delivered an SGA infant compared with 8.7% (95% CI, 8.7%-8.8%) of women without ACHD (aOR, 1.4; 95% CI, 1.2-1.6). However, results for women with several subtypes of ACHD were not significant, including for women with anomalies of venous return, anomalies of the atria and interatrial communications, and ventricular septal defects. Women with anomalies of the atrioventricular junctions and valves had significantly lower odds of having an SGA infant (aOR, 0.7; 95% CI, 0.5-0.8). Rates of SGA for infants who were born preterm (<37 weeks) also varied by subtype (Figure). Women with heterotaxy had the highest proportion of SGA infants born preterm (55.6%; 95% CI, 23.7%-83.4%). Overall, 14.9% (95% CI, 11.1%-19.8) of infants born to women with any type of ACHD who had a preterm birth were classified as SGA, which was significantly higher than the proportion born to women in the general population (10.4%; 95% CI, 10.3%-10.6%).

**Figure.**
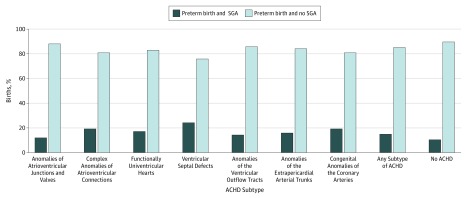
Preterm and Small-for-Gestational Age (SGA) Births to Women With Different Subtypes of Adult Congenital Heart Disease (ACHD) Preterm birth indicates less than 37 weeks’ gestation.

In infants born to women with ACHD, no time trend was observed for neonatal morbidity, preterm birth at less than 37 weeks’ gestation, or SGA births. For infants born to women without ACHD, increases over time were observed for neonatal morbidity (fiscal year 2001-2002, 2.9% [95% CI, 2.7%-3.1%]; fiscal year 2014-2015, 6.4% [95% CI, 6.3%-6.5%]; *P* < .001), preterm birth (fiscal year 2001-2002, 5.1% [95% CI, 4.8%-5.4%]; fiscal year 2014-2015, 7.4% [95% CI, 7.3%-7.5%]; *P* < .001), and SGA births (fiscal year 2001-2002, 7.7% [95% CI, 7.4%-8.1%]; fiscal year 2014-2015, 9.3% [95% CI, 9.2%-9.4%]; *P* < .001). In a sensitivity analysis in which alternative correlation structures were evaluated, no differences were observed in any of these outcomes (eTable 5 in the Supplement).

## Discussion

Our study reports 2114 births to women with ACHD, which, to our knowledge, is the largest sample established internationally. The results suggest an association between several adverse neonatal and maternal outcomes and ACHD. With the exception of major congenital anomalies, women with ACHD and their infants had significantly higher odds of all outcomes investigated; however, substantial variation in outcomes was observed by ACHD subtype. This variation suggests that women with certain types of ACHD can expect to have similar obstetric and neonatal outcomes to those observed in the general population, whereas women at highest risk can benefit from preconception counseling and close clinical monitoring during pregnancy.

Our study had similar results to previous studies on this population. For example, an Australian retrospective cohort study from 1999 to 2004^31^ reported an overall rate of severe maternal morbidity of 1.25%, compared with 1.1% in the population in our study (in that study,^31^ data were reviewed for 500 603 women, of whom 6242 had severe maternal morbidity). Similar to other large-scale studies of ACHD and pregnancy, the risk of severe maternal morbidity for women with ACHD was higher than that of women without ACHD.^32,33,34^

Similar to the results of other large cohort studies examining pregnancy and ACHD,^11,15^ women with ACHD had higher odds of experiencing a preterm birth. However, these odds were lower in our study than in other studies. In a literature review of pregnancy outcomes experienced by women with cardiac disease, Drenthen and colleagues^8^ reported an overall risk of 15.9% in a cumulative 1413 pregnancies; their analysis revealed variations in the rate of preterm birth by subtype of congenital heart disease ranging from 6.0% to 64.7%, whereas our study reports a range of 9.7% to 27.2% across subtypes. Our estimate of SGA births (12.8%) was higher than their reported 8.0% in 1381 births,^8^ but our results fit within the range reported by CHD subtype (0.0% to 66.7%). Similar to the results from Drenthen and colleagues,^8^ perinatal mortality was substantially higher in infants born to women with ACHD than those in the general obstetric population. However, our numbers may be underestimated, especially for stillbirth. Stillbirth rates in the general population are approximately 0.8%.^35^ Drenthen and colleagues^8^ reported a stillbirth rate of 2.3% for women with cardiac disease. However, in our sample, the perinatal mortality rate for the general population was only 0.2%. It is likely that the absolute risk of perinatal mortality in women with and without ACHD is higher.

Previous authors^36^ have provided hypotheses for the underlying pathophysiological mechanisms for perinatal complications observed in this population, such as preeclampsia and fetal growth restriction. The ZAHARA II study group^36^ characterized maternal cardiac indices and uterine and umbilical blood flow indices in a population of pregnant women with ACHD who, compared with controls, exhibited a relative increase in resistance in uterine blood flow at 20 and 32 weeks’ gestation. Subsequent work^37^ has further described the association between maternal right ventricular function and uterine blood flow in pregnant women with ACHD throughout pregnancy, finding that women with signs of cardiac dysfunction were more likely to demonstrate abnormal uterine blood flow. Abnormal uterine blood flow early in pregnancy was associated with an increased risk of uteroplacental complications, such as preeclampsia and fetal growth restriction.^37^

In this study, adult congenital heart disease was associated with increased and varying risks of neonatal and maternal morbidities during pregnancy. For some women with ACHD, pregnancy was not associated with substantially increased odds of maternal morbidity or neonatal morbidity, preterm birth, or mortality. Preconception and prenatal counseling for women with ACHD considering pregnancy should convey the risks of pregnancy for mother and infant and plan for pregnancy care based on maternal and perinatal risks.^38,39^ Understanding the variation in odds of adverse maternal and neonatal outcomes for women with different subtypes of ACHD is vital for effective counseling. Knowledge of these risks should be coupled with expert analysis of pregnancy risk assessment (eg, Cardiac Disease in Pregnancy,^11^ World Health Organization^40^), including the woman’s structural lesion at birth, type of repair, residual lesions, current functional status,^38^ known risk factors (eg, pulmonary hypertension), and other clinical variables. Owing to these complexities, preconception counseling, antenatal care, intrapartum care, and postpartum surveillance should be coordinated by a multidisciplinary group with expertise in managing ACHD pregnancies, especially for women with high-risk lesions.^39^

### Strengths and Limitations

This study has strengths and limitations. The use of administrative data allowed us to identify patients during birth events, because approximately 99% of births in Canada occur in a hospital setting.^41^ All Canadian citizens and permanent residents have universal provincial health insurance coverage, meaning that administrative hospitalization data cover the entire population and are not subject to selection bias. In addition, these data are coded by trained health coders in a consistent manner and are routinely monitored for accuracy. The large sample allowed us to examine rare outcomes and to analyze outcomes by ACHD subtype. In addition, the DAD represents all hospital discharges in each Canadian province (excluding Quebec) and has no sampling of discharges, thereby allowing us to gain a broader perspective of outcomes for women with ACHD who experience pregnancy rather than focusing on only a high-risk clinical sample who require specialized care, as in the previous literature.^7,10,11,13,16,17^ The main drawback of administrative data in this study is associated with the lack of clinical detail. For example, we were unable to determine the lesion severity or whether a lesion had been surgically corrected in childhood. We attempted to overcome these issues by using the Anatomic and Clinical Classification of Congenital Heart Defects scheme, which groups clinically similar lesions together for the purposes of epidemiological studies. In addition, because the available data set was limited to the women’s labor and delivery hospitalization, we may have missed other outcomes that occurred antenatally or post partum. Thus, our reported estimates are likely conservative, excluding other adverse events that would occur throughout and immediately after pregnancy. This study examined only pregnancies that continued past 20 weeks’ gestation, excluding pregnancies that may have been terminated on medical advice and pregnancy losses at less than 20 weeks; this may have underestimated the reported outcomes.

## Conclusions

To our knowledge, this is the first population-based study to systematically and simultaneously quantify the association of different forms of ACHD with obstetric and neonatal outcomes at the time of delivery. This quantification provides important clinical information for counseling patients who are pregnant or contemplating pregnancy about complications they might experience at the time of delivery. Certain ACHD subtypes may be associated with increased odds of adverse maternal and neonatal outcomes. It appears from our study that these subtypes should be monitored closely during pregnancy, and prenatal counseling provided to address these risks and any mitigation strategies.

## Notes

Supplement. **eTable 1.** Anatomic and Clinical Classification of Congenital Heart Defects (ACC-CHD) With Description and *International Statistical Classification of Diseases and Related Health Problems, Tenth Revision* (*ICD-10*), Code(s) **eTable 2.** Diagnostic and Procedural Codes for the Maternal Morbidity Outcomes Indicator (MMOI) **eTable 3.** Diagnostic and Procedural Codes for the Neonatal Adverse Outcomes Indicator (NAOI) **eTable 4.** Major Visible Congenital Anomalies and Their Diagnostic Codes **eTable 5.** Generalized Estimating Equations: Sensitivity Analyses of Potential Correlation Structures **eReferences.**
