# Supplementary material for: Association of Adult Congenital Heart Disease With Pregnancy, Maternal, and Neonatal Outcomes
Source: JAMA Netw Open. 2019 May 10;2(5):e193667. doi: 10.1001/jamanetworkopen.2019.3667 (PMC6512464; doi:10.1001/jamanetworkopen.2019.3667)
Supplement: Supplement. — eTable 1. Anatomic and Clinical Classification of Congenital Heart Defects (ACC-CHD) With Description and International Statistical Classification of Diseases and Related Health Problems, Tenth Revision (ICD-10), Code(s) eTable 2. Diagnostic and Procedural Codes for the Maternal Morbidity Outcomes Indicator (MMOI) eTable 3. Diagnostic and Procedural Codes for the Neonatal Adverse Outcomes Indicator (NAOI) eTable 4. Major Visible Congenital Anomalies and Their Diagnostic Codes eTable 5. Generalized Estimating Equations: Sensitivity Analyses of Potential Correlation Structures eReferences. [file jamanetwopen-2-e193667-s001.pdf]

Supplementary Online Content

Ramage K, Grabowska K, Silversides C, Quan H, Metcalfe A. Association of adult congenital heart disease with pregnancy, maternal, and neonatal outcomes. *JAMA Netw Open.* 2019;2(5):e193667. doi:10.1001/jamanetworkopen.2019.3667

eTable 1. Anatomic and Clinical Classification of Congenital Heart Defects (ACC-CHD) With Description and *International Statistical Classification of Diseases and Related Health Problems, Tenth Revision* (*ICD-10*), Code(s)
eTable 2. Diagnostic and Procedural Codes for the Maternal Morbidity Outcomes Indicator (MMOI)
eTable 3. Diagnostic and Procedural Codes for the Neonatal Adverse Outcomes Indicator (NAOI)
eTable 4. Major Visible Congenital Anomalies and Their Diagnostic Codes
eTable 5. Generalized Estimating Equations: Sensitivity Analyses of Potential Correlation Structures
eReferences.

This supplementary material has been provided by the authors to give readers additional information about their work.

eTable 1. Anatomic and Clinical Classification of Congenital Heart Defects (ACC-CHD) With Description and *International Statistical Classification of Diseases and Related Health Problems, Tenth Revision* (*ICD-10*), Code(s)

ACC-CHD Classification	Description	ICD-10 Code(s)
**Heterotaxy, including isomerism and mirror-imagery**	Heterotaxy syndromes (heterotaxia syndrome, visceral heterotaxy: abnormal arrangement of thoraco-abdominal organs)	Q89.3
	Isomerism of atrial appendages (left and right)	Q20.6
	Situs inversus (mirror-imaged arrangement)	Q89.3
**Anomalies of the venous return**	Anomalies of the systemic venous return (congenital):<br>• Congenital anomaly of the superior vena cava (SVC)<br>○ absent right SVC<br>○ retro-aortic innominate vein<br>○ left SVC persisting to coronary sinus<br>○ left SVC persisting to left-sided atrium<br>• Congenital anomaly of the coronary sinus<br>○ partially unroofed coronary sinus<br>○ totally unroofed coronary sinus<br>○ coronary sinus orifice atretic<br>• Congenital anomaly of the inferior vena cava (IVC)<br>○ azygos continuation of the IVC<br>○ right-sided azygos continuation of the IVC<br>○ left-sided azygos continuation of the IVC<br>• Congenital anomaly of the hepatic veins<br>○ separate hepatic and IVC venous return	 Q26.9<br>Q26.8<br>Q26.9<br>Q26.1<br>Q26.1<br>Q21.1<br>Q21.1<br>Q21.1<br>Q21.1<br>Q26.9<br>Q26.8<br>Q26.8<br>Q26.8<br>Q26.8<br>Q26.8
	Anomalies of the pulmonary venous connections:<br>• Anomalous pulmonary venous connection<br>○ totally (supracardiac, intracardiac, infracardiac, or mixed)<br>○ partially<br>• Congenital pulmonary vein stenosis<br>• Congenital pulmonary vein hypoplasia<br>• Pulmonary vein(s) atresia<br>• Scimitar syndrome	 Q26.4,<br>Q26.2,<br>Q26.3<br><br>Q26.8<br>Q26.8<br>Q26.8<br>Q26.8
**Anomalies of the atria and interatrial communications (IAC)**	Cor triatriatum:<br>• Cor triatriatum dexter (obstructive Eustachian valve)	Q24.2<br>Q24.2
	Interatrial Communications<br>• Patent oval foramen<br>• Ostium secundum type<br>• Sinus venosus type (superior)<br>• Coronary sinus type	Q21.1<br>Q21.1<br>Q21.1<br>Q21.1<br>Q21.1
	Juxtaposition of the atrial appendages (JAA)	Q20.8

©2019 Ramage K et al. *JAMA Network Open.*

ACC-CHD Classification	Description	ICD-10 Code(s)
	• Left JAA	Q20.8
	• Right JAA	Q20.8
**Anomalies of the Atrioventricular Junctions and Valves**	Congenital anomalies of the tricuspid valve (TV)	
	• Ebstein’s malformation	Q22.5
	○ associated with discordant AV connections	Q22.5 +
	• TV agenesis (unguarded tricuspid orifice)	Q20.5
	• Dysplastic tricuspid valve	
	• Congenital tricuspid insufficiency	Q22.9
	• Cleft of the TV	Q22.8
	• Congenital valvar tricuspid stenosis	Q22.8
	• Tricuspid annular hypoplasia	Q22.8
	• Straddling TV	Q22.4
	• Overriding TV	Q22.4
	• Anomaly of the TV subvalvar apparatus	Q22.8
	Congenital anomalies of the mitral valve (MV)	
	• Dysplastic MV	Q23.8
	• Congenital mitral insufficiency	Q23.3
	• Isolated cleft of the MV (not AVSD type)	Q23.9
	• Congenital MV prolapse	I34.1
	• Congenital mitral valvar stenosis	Q23.2
	• Supravalvar mitral ring	Q20.8
	• Mitral annular hyposplasia	Q23.2
	• Parachute MV	Q23.8
	• Double mitral orifice	Q23.9
	• Straddling MV	Q23.8
	• Overriding MV	Q23.8
	• Congenital anomaly of the MV subvalvar apparatus	Q23.8
	• Accessory mitral tissue	Q23.9
	Atrioventricular septal defects (AVSD)	
	• “Complete” AVSD (ventricular and atrial components with common AV orifice)	Q21.2
	○ with left ventricular (LV) hypoplasia	Q21.2 +
	○ with right ventricular (RV) hypoplasia	Q20.8
	○ with Tetratology of Fallot	
	• “Partial” AVSD	Q21.2 +
	○ ostium primum type (atrial shunting only),	Q20.8
	○ “intermediate” or “transitional” type (atrial shunting and restrictive ventricular shunting)	Q21.1 +
	○ cleft of the left atrioventricular (AV) valve in AVSD	Q21.3
	(“mitral cleft” in AVSD)	Q21.2,
		Q23.9

©2019 Ramage K et al. *JAMA Network Open.*

ACC-CHD Classification	Description	ICD-10 Code(s)
	○ common atrium (virtual absence of atrial septum)<br>○ isolated ventricular component (ventricular shunting only)	
**Complex anomalies of atrioventricular connections**	Congenitally corrected transposition of the great arteries (double discordance)	Q20.5
	Criss-cross atrioventricular connections	Q24.8
	Supero-inferior ventricles	Q24.8
**Functionally univentricular hearts**	Double-inlet ventricle (DIV)<br>• With 2 atrioventricular valves<br>○ double-inlet right ventricle<br>○ double-inlet left ventricle<br>• Right-sided AV valve in DIV atretic (imperforate)<br>• Left-sided AV valve in DIV atretic (imperforate)<br>• Common AV orifice in double-inlet ventricle	Q20.4<br>Q20.4<br><br>Q20.4<br>Q20.4<br>Q20.4
	Absence of one atrioventricular connection<br>• Absent left-sided AV connection (mitral atresia)<br>• Absent right-sided AV connection (tricuspid atresia)	 Q23.2<br>Q22.4
	Left ventricular (LV) hypoplasia<br>• Hypoplastic left hear syndrome<br>• Mitral valve atretic (imperforate)<br>• Ventricular imbalance with dominant RV and hypoplastic LV	Q20.8<br>Q23.4<br>Q23.2<br>Q20.8
	Right ventricular (RV) hypoplasia<br>• Pulmonary atresia with intact ventricular septum<br>• Tricuspid valve atretic (imperforate): congenital<br>• Hypoplastic right heart syndrome<br>• Ventricular imbalance with dominant LV and hypoplastic RV<br>• Uhl's anomaly	Q20.8<br>Q22.0<br>Q22.4<br>Q22.6<br>Q20.8<br>Q24.8
**Ventricular septal defects (VSD)**	Perimembranous VSD	Q21.0
	Perimembranous VSD, small	Q21.0
	Perimembranous VSD with posterior (inlet) extension	Q21.0
	Malalignment (infundibular, conoventricular) VSD (with malaligned outlet (conal) septum)<br>• Anterior malalignment VSD (Fallot type)<br>• Posterior malalignment VSD (aortic arch obstruction type)	Q21.0
	Doubly committed (subarterial, conal) VSD	Q21.0
	Muscular VSD	Q21.0
	Muscular VSD, small	Q21.0
	Inlet VSD, not associated with a common AV junction	Q21.0
	Multiple VSDs	Q21.0
	Associated with aortic insufficiency (Prolapsed aortic leaflet)	Q21.0

©2019 Ramage K et al. *JAMA Network Open.*

ACC-CHD Classification	Description	ICD-10 Code(s)
**Anomalies of the ventricular outflow tracts (ventriculo-arterial connections)**	Transposition of the great arteries (TGA)	
	• TGA with intact ventricular septum	Q20.3
	• TGA (discordant ventriculo-arterial connections)	Q20.3
	Other abnormal ventriculo-arterial (VA) connections	
	• Double outlet right ventricle (DORV)	Q20.1
	○ With subaortic VSD	Q20.1
	○ With sub-pulmonary VSD	Q20.1
	○ With non-committed VSD	Q20.1
	○ With doubly committed VSD	Q20.1
	○ With intact ventricular septum	Q20.1
	○ Fallot type (subaortic or double committed VSD + pulmonary stenosis)	Q20.1
	• Double outlet left ventricle	Q20.2
	• Concordant VA connections with parallel great arteries (anatomically corrected malposition of the great arteries)	Q25.9
	Tetralogy of Fallot and variants	
	• Tetralogy of Fallot (TOF)	Q21.3
	• Tetralogy of Fallot with pulmonary atresia	Q22.0 +
	○ With MAPCAs (collaterals)	Q21.0
	• Absent pulmonary valve syndrome	Q22.0 +
	○ With non-Fallot VSD	Q21.0
	○ With Fallot-type VSD	Q22.3
	Anomalies of the intrapericardial arterial trunks	
	• Common arterial trunk (truncus arteriosus)	Q20.0
	○ With common origin of PAs (type I)	Q20.0
	○ With separate origin of PAs (type II)	Q20.0
	○ With isolated (discontinuous PAs)	Q20.0
	○ With aortic arch obstruction	Q20.0
	• Aorto-pulmonary window	Q21.4
	• Pulmonary artery (PA) from ascending aorta	Q25.7
	○ Right PA from ascending aorta	Q25.7
	○ Left PA from ascending aorta	Q25.7
	• Anomalies of the ascending aorta	
	○ Atresia of the ascending aorta	Q25.2
	○ Supravalvar aortic stenosis	Q25.3
	○ Aortic sinus of Valsalva aneurysm	Q25.4
	○ Aorto-left ventricular tunnel	Q25.4
	• Supravalvar pulmonary stenosis	Q25.6
	Left ventricular outflow tract (LVOT) and aortic valvar anomalies	
	• Valvar aortic stenosis: congenital	
	• Bicuspid aortic valve	Q23.0

©2019 Ramage K et al. *JAMA Network Open.*

ACC-CHD Classification	Description	ICD-10 Code(s)
	• Dysplastic aortic valve	Q23.1
	• Aortic valve atresia	Q23.8
	• LVOT obstruction	Q23.0
	• Subvalvar aortic stenosis (fibromuscular tunnel)	Q24.4
	• Subaortic fibromuscular shelf	Q24.4
	• Congenital aortic insufficiency	Q24.4
		Q23.1
	Right ventricular outflow tract (RVOT) and pulmonary valvar anomalies	
	• Pulmonary valvar stenosis: congenital	Q22.1
	• Dysplastic pulmonary valve	Q22.3
	• Bicuspid pulmonary valve	Q22.3
	• Pulmonary atresia with other CHD (with additional code for any associated lesion(s))	Q22.0
	• Subpulmonary stenosis	Q24.3
	○ RVOT obstruction (subpulmonary)	Q24.3
	○ Stenosis of mouth of the infundibulum	Q24.3
	○ RVOT obstruction due to malaligned outlet septum	Q24.3
	• Double chambered right ventricle	Q24.8
**Anomalies of the extrapericardial arterial trunks**	Patent ductus arteriosus	Q25.0
	• Patent ductus arteriosus, atypical (abnormal origin)	Q25.8
	Coarctation of the aorta	Q25.1
	• Coarctation of the aorta, abdominal	Q25.1
	• Hypoplasia of the aortic arch (tubular)	Q25.4
	Interruption of the aortic arch	Q25.4
	• Type A: distal to subclavian artery	Q25.4
	• Type B: between subclavian and common carotid	Q25.4
	• Type C: between carotid arteries	Q25.4
	Anomalies of the aortic arches	
	• Right aortic arch	Q25.4
	• Cervical aortic arch	Q25.4
	• Aberrant origin subclavian artery	Q27.8
	○ Aberrant origin of right subclavian artery	Q27.8
	○ Aberrant origin of left subclavian artery	Q27.8
	• Arterial duct from left innominate artery	Q27.8
	• Double aortic arch	Q25.4
	• Persistent fifth aortic arch	Q25.4
	Anomalies of the pulmonary artery (PA) and its branches	
	• Pulmonary arterial stenosis	Q25.6
	○ Right PA stenosis	Q25.6
	○ Left PA stenosis	Q25.6

©2019 Ramage K et al. *JAMA Network Open.*

ACC-CHD Classification	Description	ICD-10 Code(s)
	• Pulmonary arterial hypoplasia<br>○ Right PA hypoplasia<br>○ Left PA hypoplasia<br>• Pulmonary artery absent<br>○ Right PA absent<br>○ Left PA absent<br>• Pulmonary artery from patent arterial duct<br>○ Right PA from patent arterial duct<br>○ Left PA from patent arterial duct<br>• Pulmonary arterial sling	Q25.7<br>Q25.7<br>Q25.7<br>Q25.7<br>Q25.7<br>Q25.7<br>Q25.7<br>Q25.7<br>Q25.7<br>Q25.7
**Congenital anomalies of the coronary arteries**	Anomalous origin of left coronary artery from pulmonary artery (ALCAPA)	Q24.5
	Single coronary artery supplying all the heart	Q24.5
	Coronary orifice stenosis: congenital	Q24.5
	Coronary orifice atresia	Q24.5
	Aberrant course of left anterior descending from right coronary artery across RVOT	Q24.5
	Right ventricular myocardial sinusoids	Q24.8
	Coronary artery fistula: congenital<br>• Coronary fistula to pulmonary artery	Q24.5<br>Q24.5

Adapted from Houyel et al.[1]

©2019 Ramage K et al. *JAMA Network Open.*

eTable 2. Diagnostic and Procedural Codes for the Maternal Morbidity Outcomes Indicator (MMOI)

	Maternal Morbidity Outcome	International Classification of Disease Version 10 Canadian Modification (ICD-10-CA) Codes
DIAGNOSES	Acute Abdomen	K35, K37, K65.0, K65.9, N73.3, N73.5
	Acute Renal Failure	O90.4, N17, N19, N99.0
	Acute Psychosis	F23, F53.1
	Cardiac arrest, failure or infarction	O89.1, O74.2, O90.3, I21, I42, I43, I46, I50, J81
	Cerebral oedema or coma	R40.2, G93.6
	Disseminated intravascular coagulopathy	D65
	Cerebrovascular accident	I61-I64
	Major complications of anaesthesia	O29.0-O.292, O74.0, O74.2, O74.3, O.89.0-O89.2
	Obstetric embolism	O88
	Shock	R57, O75.1, T80.5, T88.6
	Sickle cell anaemia with crisis	D57.0
	Status asthmaticus	J46
	Status epilepticus	G41
	Uterine rupture	O71.0, O71.1
PROCEDURES	Assisted ventilation including tracheostomy	1.GZ.31, 1.GJ.77
	Curettage in combination with a general anaesthetic	5.PC.91.GA, 5.PC.91.GC, 1.RM.87, 5.CA.89.GC, 5.CA.89.GA
	Dialysis	1.PZ.21, 1.CG.76, 1.OA.21
	Evacuation of haematoma	5.PC.73, 1.AA.52, 1.AB.52, 1.AB.87, 1.AF.52, 1.AN.52, 1.AX.52, 1.CZ.52, 1.DA.42, 1.EZ.52, 1.FA.52, 1.FH.52, 1.FR.52, 1.FX.52, 1.OJ.52, 1.OT.52, 1.PB.52, 1.PE.52, 1.QG.52, 1.RW.52, 1.RY.52, 1.SH.52, 1.SZ.52, 1.TX.52, 1.UY.52, 1.VX.52, 1.WV.52, q.YA.52, 1.YC.52, 1.YD.52, 1.YE.52, 1.YF.52, 1.YG.52, 1.YM.52, 1.YR.52, 1 YS.52, 1.YT.52, 1.YU.52, 1.YV.52, 1.YW.52, 1.YX.52, 1.YZ.52
	Hysterectomy	1.RM.87, 1.RM.89, 1.RM.91
	Procedures to reduce blood flow to uterus	1.RM.13
	Reclosure of disrupted caesarean section wound	5.PC.80.JK, 5.PC.80.JM, 5.PC.80.JH
	Repair of bladder or cystostomy	1.PM.80, 1.PM.52, 1.PG.50
	Repair of intestine	1.NE.80, 1.NK.80, 1.NM.80
	Repair ruptured or inverted uterus	5.PC.91.HP, 5.PC.91.HQ, 1.RM.80
	Transfusion of blood or coagulation factors	1.LZ.19

Adapted from Roberts et al.[2]

©2019 Ramage K et al. *JAMA Network Open.*

eTable 3. Diagnostic and Procedural Codes for the Neonatal Adverse Outcomes Indicator (NAOI)

	Neonatal Outcome	International Classification of Disease Version 10 Canadian Modification (ICD-10-CA) Codes
**DIAGNOSES**	Gestational age <32 weeks	-
	Birthweight <1500 grams	-
	Neonatal mortality	-
	Respiratory Distress Syndrome	P22.0
	Seizure	P90
	Intraventricular hemorrhage (grades 2, 3, or 4)	P52.1, P52.2
	Cerebral infarction	I63
	Periventricular leukomalacia	P91.2
	Birth trauma	P10-P15
	Hypoxic ischemic encephalopathy	P91.6
	Necrotising enterocolitis	P77
	Bronchopulmonary dysplasia	P27.1
	Sepsis/septicaemia	P36, A40, A41, A02.1, A22.7, A26.7, A32.7, A42.7, B37.7, O85
	Pneumonia	J12-J18, P23
	Other respiratory: primary atelectasis, respiratory failure	J96, P22, P28
**PROCEDURES**	Resuscitation	1.GZ.30
	Ventilary support (mechanical ventilation or CPAP)	1.GZ.31
	Central venous or arterial catheter	1.IS.53, 1.KV.53
	Transfusion of blood or blood products	1.LZ.19
	Pneumothorax requiring an intercostal catheter	1.GT.33

Adapted from Lain et al.3

©2019 Ramage K et al. *JAMA Network Open.*

eTable 4. Major Visible Congenital Anomalies and their Diagnostic Codes

**Major Visible Congenital Anomalies**	**International Classification of Disease Version 10 Canadian Modification (ICD-10-CA) Codes**
Anencephaly	**Q00.0**
Cleft lip/palate	**Q35.x, Q36.x, Q37.x**
Congenital diaphragmatic hernia	**Q79.0, Q79.1**
Encephalocele	**Q01.x**
Gastroschisis	**Q79.3**
Hypospadias	**Q54.x (except Q54.4)**
Omphalocele	**Q79.2**
Spina bifida	**Q05.x**
Reduction defects of upper limb	**Q71.x**
Reduction defects of lower limb	**Q72.x**
Reduction defects of unspecified limb	**Q73.x**
Trisomy 21	**Q90.x**

Adapted from Metcalfe et al.[4]

©2019 Ramage K et al. *JAMA Network Open.*

eTable 5. Generalized Estimating Equations: Sensitivity Analyses of Potential Correlation Structures

To determine the most appropriate correlation structure for use with the generalized estimating equations, we tested several of our primary outcomes using two different types of correlation structure: independent and exchangeable.

		Correlation Structure	
Outcome		Exchangeable	Independent
Maternal Morbidity Outcome Indicator (MMOI)	Unadjusted Odds Ratio (95% CI) for women with ACHD compared with women without ACHD	3.7 (3.0-4.6)*	3.7 (3.0-4.6)*
	Adjusted Odds Ratio (95% CI) for women with ACHD compared with women without ACHD	2.7 (2.2-3.4)*	2.6 (2.1-3.2)*
Neonatal Adverse Outcomes Indicator (NAOI)	Unadjusted Odds Ratio (95% CI) for infants born to women with ACHD compared with infants born to women without ACHD	2.3 (2.0-2.6)*	2.3 (2.0-2.7)*
	Adjusted Odds Ratio (95% CI) for infants born to women with ACHD compared with infants born to women without ACHD	1.8 (1.6-2.1)*	1.9 (1.6-2.2)*
Preterm Birth (<37 weeks gestation)	Unadjusted Odds Ratio (95% CI) for infants born to women with ACHD compared with infants born to women without ACHD	2.0 (1.7-2.2)*	2.0 (1.8-2.3)*
	Adjusted Odds Ratio (95% CI) for infants born to women with ACHD compared with infants born to women without ACHD	1.5 (1.3-1.8)*	1.6 (1.4-1.9)*

* denotes significance at the p<0.05 level

Adjusted models have been controlled for mode of delivery, obstetric comorbidity, and year of delivery

©2019 Ramage K et al. *JAMA Network Open.*

eReferences
1. Houyel L, Khoshnood B, Anderson RH, et al; EPICARD Study group. Population-based evaluation of a suggested anatomic and clinical classification of congenital heart defects based on the International Paediatric and Congenital Cardiac Code. Orphanet J Rare Dis. 2011;6:64. Medline:21968022
2. Roberts CL, Cameron CA, Bell JC, Algert CS, Morris JM. Measuring maternal morbidity in routinely collected health data: development and validation of a maternal morbidity outcome indicator. Med Care. 2008;46(8):786-794.
3. Lain SJ, Algert CS, Nassar N, Bowen JR, Roberts CL. Incidence of severe adverse neonatal outcomes: use of a composite indicator in a population cohort. Matern Child Health J. 2012;16(3):600-608.
4. Metcalfe A, Sibbald B, Lowry RB, Tough S, Bernier FP. Validation of congenital anomaly coding in Canada's administrative databases compared with a congenital anomaly registry. Birth Defects Res A Clin Mol Teratol. 2014;100(2):59-66.

©2019 Ramage K et al. *JAMA Network Open.*
